# The Occurrence and Contamination Level of Ochratoxin A in Plant and Animal-Derived Food Commodities

**DOI:** 10.3390/molecules26226928

**Published:** 2021-11-17

**Authors:** Xianjiang Li, Wen Ma, Zhiyong Ma, Qinghe Zhang, Hongmei Li

**Affiliations:** 1 Food Safety Laboratory, Division of Metrology in Chemistry, National Institute of Metrology, Beijing 100029, China; zhangqh@nim.ac.cn (Q.Z.); lihm@nim.ac.cn (H.L.); 2 State Key Laboratory of Natural and Biomimetic Drugs, School of Pharmaceutical Sciences, Peking University, Beijing 100191, China; wen.ma@bjmu.edu.cn; 3 Beijing State Key Laboratory of Organic-Inorganic Composites, College of Chemical Engineering, Beijing University of Chemical Technology, Beijing 100029, China; mazhy@mail.buct.edu.cn

**Keywords:** ochratoxin A, occurrence, contamination level, food commodities, risk monitoring

## Abstract

Ochratoxin A (OTA) is a highly toxic mycotoxin and poses great threat to human health. Due to its serious toxicity and widespread contamination, great efforts have been made to evaluate its human exposure. This review focuses on the OTA occurrence and contamination level in nine plant and animal derived food commodities: cereal, wine, coffee, beer, cocoa, dried fruit, spice, meat, and milk. The occurrence and contamination level varied greatly in food commodities and were affected by many factors, including spices, geography, climate, and storage conditions. Therefore, risk monitoring must be routinely implemented to ensure minimal OTA intake and food safety.

## 1. Introduction

Mycotoxins are toxic secondary metabolites produced by some fungal species [1]. As reported, more than hundreds of mycotoxins have been discovered so far [2]. Isolated in South Africa in 1965 [3], ochratoxins are one of the most common mycotoxins, produced by many kinds of *Aspergillus* and *Penicillium* fungi. In the family of ochratoxins, ochratoxin A (OTA) is the most well-known member for its high occurrence and good stability. OTA tends to survive during storage and processing, and high cooking temperatures cannot completely destroy it. Therefore, once foodstuffs are contaminated with OTA, it is very difficult to totally remove it. Therefore, pre-harvest prevention is the most effective strategy to get rid of OTA contamination and therefore lower human exposure [4].

Studies have shown that OTA may lead to multiple health issues because of its toxicological effects, such as teratogenicity, immunotoxicity, carcinogenicity, genotoxicity, neurotoxicity, and hepatotoxicity [5]. The kidney is believed to be the primary target organ, and OTA is suspected to be the main reason for Balkan Endemic Nephropathy [6]. In addition to being toxic to humans, OTA is poisonous to livestock, such as pigs, cows, goats, poultry, and so on. Especially, pigs are quite susceptible to it because OTA was proved to be a causal agent of mycotoxic porcine nephropathy reported in Denmark during the 1960–1970s [7]. OTA has a strong affinity towards proteins, which leads to bio-accumulation and carry-over effects in animal-derived products that further endanger human health. According to the International Agency for Research on Cancer, OTA has been classified in class 2B as a possible human carcinogen.

The occurrence of OTA in food commodities has been reported worldwide, and total avoidance of OTA consumption is almost impossible. There is consensus that these OTA contaminated foods are a severe risk source to public health [8]. Estimation of the occurrence level and the main contribution of contaminated food commodities to human exposure is the first step to improve the level of OTA surveillance and risk management. From a worldwide view, cereals are considered as the major food commodity for human exposure. There are also reports in a myriad of other commodities, including fruit, cocoa, spice, coffee, etc. These OTA contaminated foods can further contaminate processed food through the food chain, such as beer, wine, juice, ham, salami, cheese, and so on [9]. Reports also showed that OTA was even found in human breast milk [10]. In these surveys, OTA occurrence could be monitored by several techniques [11] like liquid chromatography-fluorescence detector (LC-FLD) [12], liquid chromatography-mass spectrometry (LC-MS) [13,14], capillary electrophoresis laser induced fluorescence [15], electrochemical biosensor [16], and enzyme-linked immunosorbent assay [17]. There have been several reviews focusing on OTA occurrence in specific commodities, including cereal derived food [18], cereal based baby food [19], wine [20], beer [21], and so on. However, there is no review covering all these food commodities in high risk of contamination.

A recent report from the European Food Safety Authority estimated the contributions of OTA exposure from dietary intake [5]. This result was in basic accordance with a former risk assessment performed by the Joint FAO/WHO Experts Committee on Food Additives that cereals ranked first, followed by wine, grape juice, roasted coffee, and pork [22]. OTA occurrence in food has been described worldwide. The aim of this review is to provide a broad overview of the OTA occurrence and contamination level in widespread plant and animal derived food commodities. This will benefit the estimation of the main contribution of contaminated foodstuffs in human exposure and improve OTA risk management. Moreover, possible factors affecting OTA contamination are summarized to address concern about food quality control during processing and storage.

## 2. Occurrence of OTA in Plant-Derived Food Commodities

### 2.1. OTA in Cereals

Cereals (rice, wheat, corn, rye, barley, oats, millet, etc.) and their products are staple food in human diets around the world. Due to climatic conditions and storage practices, they easily suffer from fungal growth and OTA contamination, and are therefore the main sources of human exposure [18]. As shown in Table 1, OTA was detected in the regions of Asia, North America, Africa, and Europe. The environmental conditions of humidity, temperature, and water activity are essential factors for the production of OTA during the time of harvest, drying, and storage of crops. A ten-year global survey showed that 15% of the tested feed samples were positive [23]. In this survey, a total of 74,821 samples were collected from 100 nations in the period from 2008 to 2017. The highest contamination occurred in South Asia, where 60.4% samples were positive. Surprisingly, the highest contamination concentration was 2000 μg/kg. China has the biggest rice production yield in the world. An analysis of 370 rice samples from six provinces in China showed 4.9% of the samples were contaminated with OTA, and only 1 sample was above 3 μg/kg, which is the maximum residue limit (MRL) of the European Commission (EC) [24]. However, the contamination was severe in some other areas. In Pakistan, researchers found that 75% of corn and wheat products were contaminated with levels over the MRL of EC [25]. The highest level even reached 360 μg/kg in one biscuit sample, which was far beyond MRL and very dangerous to health. In the last year, 46 maize samples had detected concentrations from 2.14 to 214 μg/kg [26]. The occurrence even reached 71%, and the highest level was found on Ayub-2/27 grains. This result indicated that maize varieties had significant impact on OTA occurrence. The other two studies collected samples from the Punjab region of Pakistan. The former report collected rice, corn, and corn products during 2011–2012 [27]. The contamination rate was 32%, and rice had higher occurrence than corn. The latter report focused on 208 rice samples [28]. The maximum level was 24.9 μg/kg, which was above the MRL of EC. Another survey from Iran indicated that 41% of the analyzed cereal-based baby foods were contaminated with OTA, and 7.8% were higher than the MRL of EC [29]. Moreover, rice was usually more contaminated than wheat in Iran [30].

In the United States, a survey showed that OTA was detected in 12.2% of tested barley, durum, and hard red spring wheat samples [31]. Among them, one barley and four wheat samples exceeded 5 μg/kg. None of the positive samples were from any specific region. Another survey analyzed 144 corn, oat, wheat, and rice samples [32]. More than half (52%) of the samples were contaminated in the range from 0.10 to 7.43 μg/kg. Moreover, organic samples exhibited higher contamination than conventional samples. However, other reports had inconsistent results for infant foods [33] and breakfast cereals [34] from the USA. No significant difference was observed in the tested 155 infant cereal products and 489 breakfast cereals. However, oat-based cereal had the highest frequency and contamination levels compared with corn-, rice-, and wheat-based cereal.

From a survey in Canada, five wheat samples (2.2%) were detected with OTA and OTB, and only three samples exceeded 5 μg/kg [35]. For Algeria, the situation was worse, with 62 of 81 wheat samples contaminated with OTA ranging from 0.84 to 34.75 μg/kg [36]. Researchers also found the flour manufacturing process had little impact on OTA elimination. In Portugal, 50% of the processed cereal-based foods were contaminated with OTA [37]. However, the maximum level was only 0.23 μg/kg, and all samples were below the MRL of EC. Notably, more OTA may be introduced to wheat flour during the scouring process [18]. A total of 114 samples (wheat, buckwheat, rye, oat, barley, rice, millet, and corn flour) were collected in a Serbian market from 2012 to 2016 [38]. OTA was found in 75.0% rye and 20.0% wheat, but there was none in rice and millet flours. In Poland, only 3% of rye samples were contaminated with concentrations below 2.75 μg/kg [39]. Above all, the levels of OTA in cereals vary greatly in countries and cereal commodities.

### 2.2. OTA in Wine

After cereals, wine is the second most important source of OTA in total dietary intake in Europe [40]. Damaged grapes are susceptible to be infected with ochratoxigenic fungal, and the high sugar matrix provides a perfect medium for OTA production. Contamination seems to occur in the vineyard, and many factors have been reported to affect OTA levels in wine, including geographical and climate factors. Europe is a big market for wine production and consumption. Therefore, the EC has set maximum OTA levels in wine at 2 μg/L in Regulation 1881/2006 [41]. The frequency of OTA in wines is quite extensive, but rarely exceeds this MRL, as seen in Table 2.

As the largest wine producing country, Italy cares much about OTA in its wine products and collected three continuous survey samples from Sicily. In the first survey, 96.6% of wines produced in 2007–2011 and were contaminated by OTA, with an average concentration of 0.246 μg/L [42]. In the second survey, 72.7% of wines produced in 2012–2013 and were contaminated, with the average concentration of 0.255 μg/L [43]. The third survey collected 470 wine samples, and only one positive sample was found positive by immunoenzymatic assay [44]. However, none of the tested samples exceeded the MRL of EC. Possible reasons were the warmer climate and long drying time before fermentation. These results confirmed the good quality of Sicilian wine. In the Rioja Alavesa region of Spain, 100 wine samples produced between 2005 and 2008 were tested in a survey [45]. Among them, 57 positive samples were found with OTA concentrations in the range of 0.004–0.179 μg/L. All samples were also below the MRL of EC. In Portugal, OTA was detected in 12 samples from 60 wines, and only one white wine exceeded 2 μg/L [46]. Another recent investigation showed that 3 of 92 wine samples were contaminated with OTA with concentrations below 1 μg/L in Portugal [47]. These samples covered the time period from 1984 to 2017, and two positive samples were home produced. A recent survey focused on Madeira wines [48]. None of the analyzed 30 samples exceeded the MRL of EC, with the highest concentration being 0.45 μg/kg. From the above results, OTA contamination was quite low in Portugal. Greece carried out an OTA contamination survey covering 80% of the total wine producing area [49]. Among the tested 150 samples (123 dry wines and 27 dessert wines), 69% of samples were reported with positive results. For the contaminated samples, 91% were below 1 μg/L, 9% were between 1 and 2 μg/L, and only one sample exceeded the legal level. In addition, there was no significant difference in production years from 1999 to 2006. Nevertheless, higher frequency and contamination were found in dessert wines than dry wines. More serious contamination was found in Croatia [50]. From 110 collected red wines, 98.2% were contaminated, with an average concentration of 0.040 μg/L. Wine from the southern Dalmatian islands, where it was believed to more favorable for ochratoxigenic species growth, had higher OTA content. A report from Fruška Gora of Serbia collected 113 dry wines from 2011 to 2016 [51]. Trace OTA was found in 64% of red wines, 42.6% of white wines, and 36.4% of rosé wines. The highest occurrence rate (88.9%) was in 2014 wine, produced when there was an extreme number of rainy days. In a recent report from Spain, 47% of wine samples were contaminated in the range of 0.77–2.44 μg/L [52]. OTA and OTB were screened in 59 archived Tokaj wines by on-line extraction coupled liquid chromatography [53]. Only four samples were positive for OTA, with a maximum of 1.2 μg/L.

In the United States, OTA contamination was found in more than 85% of the 41 wines evaluated [54]. Among them, 68% of samples were above 1 μg/L and only two wines were above 2 μg/L. In South America, Chile did a survey with 1188 samples covering 1000 km distance [55]. Only in 2.9% of all tested samples were OTA levels positive, with the highest concentration being 0.35 μg/L. Furthermore, the frequency of OTA in red wine (3.5%) was twice that in white wine (1.7%) due to the longer maceration time that favored the OTA transfer from grape skin to wine during the wine manufacturing process. All in all, the contamination in Chile was quite lower than other reported areas in view of this report. Another study was carried out in China for exposure assessment. From the results of 223 wine samples from 7 provinces, the concentrations varied from 0.01 to 0.98 μg/L, with an average of 0.15 μg/L [56]. Specifically, wine from Jilin province had the lowest mean value for its high latitude and low temperature. Another survey covered four provinces in China. OTA detection rate was slightly higher than in a former report: 66% for red wine and 55% for white wine [57]. The average content was 0.61 μg/L. Another survey collected 42 samples from the Hexi Corridor Region of China [58]. Results showed good quality of these samples, with a maximum of 1.27 μg/L. Researchers also found OTA coexisted with other mycotoxins, including cyclopiazonic acid, mycophenolic acid, and zearalenone.

### 2.3. OTA in Coffee

Coffee, as the third largest source of exposure, accounts for approximately 9% of the total intake of OTA in Europe [59]. The EC has set an MRL for OTA in coffee at 5 μg/kg. Coffee trees are planted in tropical areas, but coffees are roasted worldwide. Both the plant environment and processing conditions have a significant effect on the OTA concentration. Most surveys were carried out in consumer markets other than in the areas where the coffee originated. In a survey of Portuguese markets, 40 soluble coffee and coffee substitute samples were collected and analyzed by LC-FLD [60]. The maximum concentration was found in a soluble coffee, with 11.8 μg/kg. Researchers also found brand effects such that own-brand coffee samples had higher OTA concentrations than the branded ones. In a later survey in Portugal, 3 of 11 roasted coffee samples were positive [61]. The mean concentrations were 1.84 and 1.45 μg/kg for roasted and ground roasted coffee. From another report, the situation was better in Spain. The frequency was of OTA was 49% (35 of 72) in tested ground roasted coffee, but all tested samples were below the MRL of EC. [62]. Average content was 2.17 μg/kg in the range of 1.21–4.21 μg/kg. From another report concerning French roasted coffee, all 30 samples were contaminated [63]. OTA concentration ranged from trace to 11.9 μg/kg. One sample even exceeded the MRL of EC. In Italy, a survey covered 44 soluble coffees and 6 coffee products, in which 48 positive samples were found with OTA ranging from 0.32 to 6.40 μg/kg, with a mean of 1.27 μg/kg [64]. This survey also reported that no significant difference was found between normal and decaffeinated instant coffee. Subsequently, 103 coffee samples were collected from Czech markets [65]. The results showed that 71% of roasted coffee samples were positive in the range of 0.2–2.5 μg/kg, and all the instant coffee samples were positive with concentrations between 0.6 and 12.8 μg/kg. The above reports show that OTA frequently contaminated coffee in Europe. Similar situations were found in South America. In Argentina, 69% of tested coffees were contaminated with OTA [66]. The median value was 2.7 μg/kg for green coffee, 0.24 mg/kg for ground roasted coffee, and 0.43 mg/kg for soluble coffee. This indicated that the roasting process reduced OTA content in coffee. Chile carried out a survey that found all of the coffee samples positive for OTA, and 3 of 63 samples were over the MRL of EC [67]. The mean concentration for roasted coffee was 0.47 μg/kg and for instant coffee was 1.8 μg/kg. Fermented coffee was collected and analyzed in Brazil [68]. Three out of fourteen samples were positive with a maximum of 0.87 μg/kg. This indicated that the fermentation process should not be a concern for OTA exposure. Green coffee was collected from nine countries in 2015 and 2016 for analysis of 31 mycotoxins, including Vietnam (*n* = 24), Brazil (*n* = 19), Colombia (*n* = 7), Ethiopia (*n* = 5), Ivory Coast (*n* = 4), China (*n* = 4), Indonesia (*n* = 4), Mexico (*n* = 2), and Guatemala (*n* = 2) [69]. Results from LC-MS showed that OTA occurrence was 28%, with an average concentration of 1.3 μg/kg. Co-occurrence of OTA with other mycotoxins was rare.

### 2.4. OTA in Beer

Beer is widely consumed around the world. As a beverage derived from cereals, OTA may be transferred from contaminated cereals (Table 3). In Italy, a survey covered 30 brands of beer [70]. The contamination percentage was 16.7%, with an average of 0.35 μg/kg. All positive samples were below 2 μg/kg. In the Czech Republic, 44 lager beers (90%) were contaminated by OTA [71]. Among the positive beer samples, most of the OTA concentrations were below 0.1 μg/L, with only one special pepper beer exceeding 1.2 μg/L. It is possible that contaminated pepper was used in the beer production. In the Coimbra region of Portugal, 84 homemade, craft, and industrial beers were bought from market [72]. The overall occurrence of OTA was 10.6% with concentrations below 11.25 μg/L. Results showed that homemade beers presented higher contamination levels and industrial beers showed higher occurrence. Whereas in a report from China, no occurrence was found in 20 tested beer samples [73]. The detection limit of the method was 0.006 μg/L. Another survey collected 40 beer samples from 2017 to 2018 in Spain, and 20% were contaminated with 1.83 μg/L [52].

### 2.5. OTA in Cocoa

Cocoa is an important ingredient in several kinds of foods, especially chocolate. OTA is a major mycotoxin occurring in cocoa. Cocoa is produced in tropical areas. During the processing and storage stages, the beans become susceptible to contamination by fungi and OTA. As a result, the EC has set an MRL for OTA in cocoa at 2 μg/kg. Western Africa is the most important production area, but there are only a few reports about OTA occurrence in cocoa. In 2008, a survey focused on the OTA in cocoa beans from Nigeria [74]. More than 90% of the analyzed samples were positive, with concentrations ranging from trace to 0.28 μg/kg. Latin America is the third biggest production area of cocoa, and there are two reports concerning Brazilian cocoa beans. From a report in 2011, 54 samples were collected [75]. Only four samples were above the MRL of EC, and the rest (92.5%) were below the limit. The other report was published in 2019 and the frequency was similar [76]. OTA was present in 28 of 123 analyzed samples ranging from 0.25 to 7.2 μg/kg. The mean level of OTA was 1.2 μg/kg. From the Italian markets, 60% of the collected cocoa and chocolate products were positive for OTA [77]. All of the 40 cocoa powder samples were contaminated, with the concentration ranging from 0.18 to 1.82 μg/kg below the MRL of EC. A total of 130 chocolate products in Turkey were tested for OTA, with an occurrence of 24.6% [78]. OTA was more frequently found in bitter chocolate than in milk chocolate and chocolate wafers, perhaps due to the high amount of cocoa in it. In Canadian market, the frequency of occurrence was quite high. One survey reported 100% for all 32 cocoa powder and chocolate samples [79]. Another reported 89.4% for cocoa products analyzed in 2011–2012 [80]. On the basis of the risk assessment, the exposure to OTA due to the consumption of cocoa and chocolate products is not a major concern.

### 2.6. OTA in Fruits and Nuts

Fruits are one of the more nutritious and consumed foods around the world, yet OTA contamination is not a negligible issue in them. Grapes are the raw material in wine, and OTA contamination in dried wine fruit is usually much higher than that in wine [81]. In Iran, 66 samples were collected during 2012 and 2013, and LC-FLD results showed that 23 (57.5%) currant, 10 (62.5%) sultana, and 6 (60%) raisin samples were contaminated with OTA [82]. All samples were below 8.4 μg/kg, with a mean value of 2.98 μg/kg. In Pakistan, 72 fruit and dried fruit samples were bought from Punjab and Khyber Pakhtunkhwa, including fig, apricot, dried apricot, plum, and raisin [83]. The average frequency of OTA occurrence was 25%, with a mean concentration of 3.58 μg/kg. In addition, four samples were above the recommended limit of 10 μg/kg. Usually, dried fruits were more contaminated with OTA because of water loss and lengthy storage. The highest contamination concentration discussed in this paper happened in Egypt [84]. The OTA level of tested dried date palm reached 6070 μg/kg, with a median value of 58.7 μg/kg, which was far above the MRL. This may have been caused by improper storage conditions. In contrast, only one of 19 collected palm dates from Tunisia and Algeria was positive, with a concentration of 0.75 μg/kg [85]. The nation of production may have a significant effect on OTA residual. OTA was detected in 93% (37/40) raisin samples from the United States [86]. The detected concentrations ranged from 0.06 to 11.4 μg/kg, and one sample surpassed the MRL of EC. In China, the occurrence was a little lower. A survey covered 253 samples including walnuts (35), chestnuts (33), hazelnuts (20), pine nuts (20), almonds (25), dried figs (20), dried longans (15), dried jujubes (35), raisins (30), and dried persimmons (20) [87]. Only two dried persimmons and two raisins were positive, with an average concentration of 6.23 μg/kg. Another survey collected 195 raisins from 9 provinces, and only 3 samples were contaminated, with levels of 0.18–10.14 μg/kg [88]. The situation was worse for dried jujube [89]. All 20 collected samples were contaminated, with a maximum concentration of 0.18 μg/kg.

### 2.7. OTA in Spices

Various spices are commonly consumed as food. Even though their consumption quantity is not comparable with the previously discussed foods, the OTA contamination is more serious because of their processing and storage conditions. The MRLs of OTA in spices were 15–20 μg/kg according to Regulation 1881/2006 of EC. It is well-known that chili is widely used for imparting pungency and creating aroma in cooked food. Pakistan, as the third major producer, carried out a survey for natural occurrence of OTA in 2013 [90]. Among 170 chili samples, OTA was positive with the ratio of 34.7%, including 9 chili sauce, 25 crushed chili, and 25 chili powder samples, with a highest concentration of 64.5 μg/kg. A recent survey by the same lab provided worse results for chili sauce from Pakistan [91]. A total of 252 samples were collected, including red chili sauce, green chili sauce, and garlic red chili sauce samples. The frequency was two times higher than found in the former report. The highest OTA concentration detected was 114 μg/kg. Eighty dried chili samples were collected from the open markets in Malaysia and 81.25% were found to be contaminated with a mean concentration of 7.15 μg/kg [92]. In 15% of samples, OTA exceeded 10 μg/kg, with a maximum concentration of 101.2 μg/kg. In an Italian market, 31 of 130 (23.8%) samples were contaminated with OTA. Additionally, a higher frequency of OTA was found in chili than in pepper [93]. In Lebanon, the occurrence was a little higher, 30%, in allspice, pepper, chili, cinnamon, ginger, and mixture samples [94]. This result highlighted the problem of OTA contamination in this country. From a Turkish market, 105 spice samples were checked in 2012 (24 red chili flake, 22 red chili powder, 23 black pepper powder, 19 cumin, and 17 cinnamon powder samples) [95]. The frequency of OTA occurrence was 75% for red chili flake, 54.5% for red chili powder, 17.4% for black pepper powder, and 5.3% for cumin. Another recent Turkish survey showed a worse result that 87.1% of the packed and 100% of the unpacked red pepper flake samples were positive [96]. Approximately 480 kinds of Chinese spice products, including chili, pepper, prickly, cinnamon, aniseed, fennel, curry, and cumin, were collected for OTA screening in 2014 [97]. In the final report, the contamination rate was 9.6%, with concentrations in the range 0.31–30.73 μg/kg. Ginger is a root crop and widely used as a spice. A total of 120 ginger samples were collected in Nigeria, of which 89 were collected in the dry season and 31 in the rainy season [98]. Higher contamination was found during the rainy season (77%, 3.94 μg/kg) than the dry season (37%, 1.02 μg/kg). *Astragalus propinquus* root is a kind of herbal food supplement and easily contaminated by OTA. All 40 samples from a Czech market were positive with a mean concentration of 451.0 μg/kg [99]. The maximum concentration even reached 1700 μg/kg.

## 3. Occurrence of OTA in Animal-Derived Food Commodities

### 3.1. OTA in Meat and Meat Products

Animal feeds contain cereal ingredients, and OTA may transfer to and accumulate in these animals through the food chain (Table 4). Health concern always exist from consumption of animal-derived food products, especially pork [9]. However, the MRLs of OTA in meat and derivative products were not set in Regulation 1881/2006 of EC and GB 2761 of China. According to a study of OTA distribution in pig tissues, its concentration followed the order: blood plasma > lung > kidney > heart > bile > liver > fat > muscle [100]. From 2014 to 2016, Poland executed an official monitoring of OTA in pig tissues (muscle, liver, and kidney) [101]. Results showed that approximately 23.5% of the animal tissue samples were contaminated, with a mean of 2.0 μg/kg. A French survey tested muscle and liver samples from 70 pigs [102]. In this survey, OTA was quantified in 36% (*n* = 25) of the collected liver samples. Moreover, good agreement was found that OTA levels in liver were 2.9 times higher than those in muscle. In 2018, muscle, liver, and kidney samples were collected from 48 wild boars in Italy [103]. The highest concentrations of OTA were found in the kidneys. In 10 kidney and 6 liver samples, the concentrations of OTA were higher than 1 μg/kg, but all of the muscle samples were below this concentration. OTA appeared to be stable in pork-derived products. Among 110 dry-cured hams from the Italian market, OTA was found to be much lower in the inner core, with a median value below 0.1 μg/kg, than in the surface portion, with a median value of 0.53 μg/kg [104]. Salami is a typical Italian sausage made with pig muscle. A survey showed that 5 of 50 salami samples from Veneto were positive [105]. A recent study collected 172 different salamis from four Italian regions [106]. OTA was reported in 22 salamis, and 3 samples exceeded 1 μg/kg. In particular, occurrence was higher in spicy salamis, but that may be ascribed to OTA contaminated red chili pepper.

### 3.2. OTA in Milk and Milk Products

OTA was also reported in milk products as a consequence of intake of dietary OTA by livestock. The MRLs for OTA in infant formulae was 0.025 μg/kg in Regulation 1881/2006 of EC. In a report, 83 bovine, goat, and sheep milk samples (63 organic and 20 conventional milk) from Italy were tested and three samples were positive, with concentrations ranging from 0.07 to 0.11 μg/kg [107]. All three positive samples were organic milk products. In Teramo, Italy, a survey collected 33 jenny milk samples to evaluate OTA exposure in 2020 [108]. The frequency was 36.4%, with a median concentration of 7.5 ng/L. Cheese is a typical dairy product and is very popular in western world. Forty cheese samples were collected from Italy [109]. Results showed the contamination was related with the production area of cheese. Higher frequency was found in Italian cheese and no contamination was from German cheese. The overall concentration was in the range of 0.1 to 3.0 μg/kg.

Breast milk is considered as an essential food for infants, who are more susceptible to OTA adverse effects. Biasucci and coworkers collected 57 breast milk in “G. da Saliceto” Hospital in Piacenza [110]. No significant difference was found between Italian and non-Italian pregnant women. The OTA occurrence was 78.8%, with an average concentration of 10 ng/L. Additionally, 90 breast milk samples were collected in Iran and analyzed by enzyme-linked immunosorbent assay [17]. All samples were below the detection limit (5 ng/L). OTA was detectable in 79% of the breast milk samples from Chilean mothers, with average of 52 ng/L [111]. Researchers also found that OTA in first milk was 2.5 times higher than in mature milk, and OTA in plasma was four times higher than that in breast milk. The contamination was severe in Morocco. The occurrence was 55% among 82 breast milk samples with a maximum concentration of 10.04 μg/L [112].

All in all, the contribution of milk to the intake of OTA in humans is negligible compared to other food commodities such as cereals, dried fruits, wine, cocoa, and coffee.

## 4. Conclusions

As one of the most important mycotoxins, OTA contamination has been a global problem because of high toxicity, widespread existence, and lengthy persistence. This manuscript provides a view of OTA occurrence in various food commodities throughout the world. Geography and climate environment have an effect on OTA occurrence in different spices, especially in tropical areas where high temperature and moisture favor the growth of ochratoxigenic fungi. Improper storage will magnify the OTA contamination level by even thousands of times. Brand effect is obvious for some samples, maybe due to more rigorous quality control.

Therefore, monitoring programs and surveillance strategies must be routinely implemented to ensure minimal OTA exposure and guarantee food safety. In this review, the OTA occurrence and contamination levels were summarized according to the food commodities in order of exposure risk. These data will provide evidence to estimate the OTA daily intake and evaluate the exposure risk. Scientific evidence has proved the significance of combined exposure and the importance of co-occurrence of multiple mycotoxins. Hence, greater effort should be made to investigate the combined risk of multiple mycotoxin exposure following accurate dose-response relationships.

## Figures and Tables

**Table 1:** OTA occurrence and contamination level in cereals.

Matrix	Nation	Year of Production	No. of Samples	Occurrence (%)	Maximum (μg/kg)	Mean (μg/kg)	Reference
Wheat	100 nations	2008–2017	74,821	15	2000	/	[23]
Rice	China	2009–2011	370	4.9	3.2	0.85	[24]
Corn, wheat	Pakistan	2015	40	27.5	360	/	[25]
Maize	Pakistan	2016–2017	46	71	218.25	/	[26]
Rice, corn, and corn product	Pakistan	2011–2012	275	32	/	/	[27]
Rice	Pakistan	/	208	19	24.9	/	[28]
Cereal based baby foods	Iran	2017–2018	64	41	1.1	0.42	[29]
Rice	Iran	/	308	9.4	11.4	/	[30]
Barley and wheat	USA	2011–2012	262	12.2	185.24	/	[31]
Corn, oat, wheat, and rice	USA	2012–2013	144	53	7.43	0.61	[32]
Infant cereal	USA	2012–2014	155	30	22.1	/	[33]
Corn-, rice-, wheat-, and oat-based breakfast cereal	USA	2012–2014	489	41	9.3	/	[34]
Wheat	Canada	2011–2014	232	2.2	/	14.7	[35]
Wheat and derived samples	Algeria	2012–2013	81	76.65	34.75	/	[36]
Cereal based foods	Portugal	2015	20	50	0.263	0.061	[37]
Flour	Serbia	2012–2016	114	29	23.04	0.46	[38]
Rye	Poland	2017–2019	60	3	2.75	/	[39]

**Table 2:** OTA occurrence and contamination levels in wine.

Matrix	Nation	Year of Production	No. of Sample	Occurrence (%)	Maximum (μg/L)	Mean (μg/L)	Reference
Sweet wine	Italy	2007–2011	30	96.6	1.56	0.246	[42]
Red and white wine	Italy	2012–2013	100	72.7	0.711	0.255	[43]
Wine	Italy	/	470	0.2	/	/	[44]
Red wine	Spain	1995–2008	100	57	0.179	0.035	[45]
Red and white wine	Portugal	/	60	20	2.4	/	[46]
Red and white wine	Portugal	1984–2017	92	3.2	/	/	[47]
Wine	Portugal	2010	30	6.7	0.45	0.42	[48]
Red, rosé, and white wine must	Greece	1999–2006	150	69.4	2	0.26	[49]
Red wine	Croatia	2011–2015	110	98.2	0.163	0.040	[50]
Red, rosé, and white wine	Serbia	2011–2016	113	52.2	0.134	0.026	[51]
Wine	Spain	2017–2018	40	47	2.28	1.13	[52]
Tokaj wines	Slovak	1959–2017	59	6.8	1.2	/	[53]
Red and white wine	USA	2010–2015	41	85.4	8.6	1.3	[54]
Red and white wine	Chile	2007–2009	1188	2.9	0.35	/	[55]
Red, rosé, and white wine	China	/	223	45.2	0.98	0.15	[56]
Red and white wine	China	/	70	62.8	/	0.61	[57]
Wine	China	2007–2016	42	4.8	1.27	1.27	[58]

**Table 3:** OTA occurrence and contamination level in other plant-derived food.

Matrix	Nation	Year of Production	No. of Sample	Occurrence (%)	Maximum (μg/kg) *	Mean (μg/kg) *	Reference
Soluble coffee and coffee substitutes	Portugal	2012	40	87.5	11.8	/	[60]
Roasted coffee	Portugal	/	11	27.3	10.31	1.13	[61]
Roasted coffee	Spain	2008	72	48.6	4.21	2.17	[62]
Roasted coffee	France	/	30	100	11.9	/	[63]
Coffee and coffee products	Italy	2011	50	96	6.4	/	[64]
Roasted and instant coffee	Czech	2016–2018	103	80.6	12.8	/	[65]
Coffee bean roasted coffee and soluble coffee	Argentina	/	51	69	20.3	/	[66]
Roasted and instant coffee	Chile	/	63	33	7.25	1.3	[67]
Fermented coffees	Brazil	2017	14	21.4	0.87	0.18	[68]
Green coffee	9 countries	2015–2016	71	28	12.2	1.3	[69]
Beer	Italy	/	30	16.6	/	0.35	[70]
Lager beer	Czech	/	49	90	1.2 μg/L	0.06 μg/L	[71]
Lager and ale beer	Portugal	2018	84	10.6	11.25	3.14	[72]
Beer	China	2008–2009	20	0	0	0	[73]
Beer	Spain	2017–2018	40	20	3.38	1.83	[52]
Cocoa bean	Nigeria	/	59	90	0.28	/	[74]
Cocoa bean	Brazil	2006	54	92.5	4	0.45	[75]
Cocoa bean	Brazil	2015–2017	123	22.8	7.2	1.2	[76]
Cocoa and chocolate	Italy	/	300	59.7	1.82	/	[77]
Chocolate products	Turkey	2017	130	24.6	0.75	/	[78]
Cocoa and chocolate	Canada	/	60	100	7.8	0.95	[79]
Cocoa products	Canada	2011–2012	85	89.4	4.72	0.66	[80]
Dried grapes	Iran	2012–2013	66	40.9	8.4	2.98	[81]
Fruit and dried fruit	Pakistan	2016–2017	72	18	18.5	3.58	[82]
Palm dates	Egypt	2016	28	11	6070	/	[83]
Palm dates	Tunisia and Algeria	2018	19	5.3	0.75	0.75	[84]
Raisin	USA	/	40	93	11.4	0.7	[85]
Dried fruit and nuts	China	/	253	1.6	9.39	6.23	[86]
Grapes, juice, and raisin	China	2016	556	8.4	10.14	/	[87]
Dried jujube	China	2013	20	100	0.18	0.14	[88]
Chili	Pakistan	/	170	34.7	64.5	/	[89]
Chili sauce	Pakistan	2018	252	71	114	/	[90]
Dried chili	Malaysia	2009	80	81.25	101.2	7.15	[91]
Chili and pepper	Italy	2011–2012	130	23.8	19.06	6.18	[92]
Allspice, pepper, chili, cinnamon, ginger, and mixture	Italy	/	94	30	34	7.1	[93]
Chili flake, chili powder, black pepper powder, cumin, and cinnamon	Turkey	2010–2011	105	24.7	98.2	5.7	[94]
Red pepper flakes	Turkey	2012–2013	75	94.6	31.7	3.5	[95]
Pepper, chili, prickly ash, cinnamon, aniseed, fennel, curry powder, and cumin	China	2009	480	9.6	30.73	/	[96]
Ginger	Nigeria	2014	120	47.5	12.02	1.77	[97]
Astragalus propinquus	Czech	2015–2016	40	100	1700	451	[98]

* The default unit is μg/kg except for some values with units.

**Table 4:** OTA occurrence and contamination level in animal-derived food.

Matrix	Nation	Year of Production	No. of Sample	Occurrence (%)	Maximum (μg/kg) *	Mean (μg/kg) *	Reference
Swine kidney liver and muscle	Poland	2014–2016	430	23.5	/	2	[101]
Swine liver and muscle	France	2014	70	64.3	3.65	0.15	[102]
Wild boars	Italy	2014–2015	48	/	3.23	/	[103]
Dry-cured hams	Italy	/	110	76.4	5.64	/	[104]
Salami	Italy	2013	50	10	103.69	/	[105]
Salami	Italy	2015–2016	172	12.8	5.66	0.51	[106]
Bovine, goat, and sheep milk	Italy	/	83	3.6	0.11	/	[107]
Jenny milk	Italy	2020	33	36.4	82 ng/L	/	[108]
Hard cheese	Italy	2011	40	15	54.07	14.94	[109]
Breast milk	Italy	2007	57	78.8	75.1 ng/L	10 ng/L	[110]
Breast milk	Iran	2019	90	0	0	0	[17]
Breast milk	Chile	2008–2010	50	79	186 ng/L	52 ng/L	[111]
Breast milk	Morocco	2017	82	55	10.04 μg/L	2.17	[112]

* The default unit is μg/kg except for some values with units.

## Abbreviations

EC	European Commission
MRL	Maximum residue limit
OTA	Ochratoxin A
